# Retrospective analysis of amantadine response and predictive factors in intensive care unit patients with non-traumatic disorders of consciousness

**DOI:** 10.3389/fneur.2024.1512227

**Published:** 2025-01-06

**Authors:** Corinna Blum, Constanze Single, Kornelia Laichinger, Anna Hofmann, Tim W. Rattay, Kamaldeen Adeyemi, Reimer Riessen, Michael Haap, Helene Häberle, Ulf Ziemann, Annerose Mengel, Katharina Feil

**Affiliations:** ^1^Department of Neurology & Stroke, University Hospital Tübingen, Tübingen, Germany; ^2^Department of Neurology/Neurodegenerative Diseases, University Hospital Tübingen, Tübingen, Germany; ^3^Department of Neurology, University Hospital Schleswig-Holstein, Kiel, Germany; ^4^Department for Internal Medicine, University Hospital Tübingen, Tübingen, Germany; ^5^Department of Anesthesiology and Intensive Care Medicine, University Hospital Tübingen, Tübingen, Germany

**Keywords:** neurointensive care, amantadine, disorders of consciousness, non-traumatic brain disorders, response profile, adverse events, functional recovery, retrospective analysis

## Abstract

**Background:**

Disorders of consciousness (DoC) in non-traumatic ICU-patients are often treated with amantadine, although evidence supporting its efficacy is limited.

**Methods:**

This retrospective study analyzed non-traumatic DoC-patients treated with amantadine between January 2016 and June 2021. Data on patient demographics, clinical characteristics, treatment specifications, and outcomes were extracted from electronic medical records. Patients were classified as responders if their Glasgow Coma Scale (GCS) improved by ≥3 points within 5 days. Good outcome was defined as a modified Rankin Scale (mRS) of 0–2. Machine learning techniques were used to predict response to treatment.

**Results:**

Of 442 patients (mean age 73.2 ± 10.7 years, 41.0% female), 267 (60.4%) were responders. Baseline characteristics were similar between groups, except that responders had lower baseline GCS (7 [IQR 5–9] vs. 8 [IQR 5–10], *p* = 0.030), better premorbid mRS (2 [IQR 1–2] vs. 2 [IQR 1–3], *p* < 0.001) and fewer pathological cerebral imaging findings (45.7% vs. 61.1%, OR 0.56, 95% CI: 0.36–0.86, *p* = 0.008). Responders exhibited significantly lower mortality at discharge (13.5% vs. 27.4%, OR 0.41, 95% CI: 0.25–0.67, *p* < 0.001) and follow-up (16.9% vs. 32.0%, OR 0.43, 95% CI: 0.24–0.77, *p* = 0.002). Good outcomes were more frequent in responders at follow-up (4.9% vs. 1.1%, OR 6.14, 95% CI: 1.35–28.01, *p* = 0.004). In multivariate analysis higher premorbid mRS (OR 0.719, 95% CI 0.590–0.875, *p* < 0.001), pathological imaging results (OR 0.546, 95% CI 0.342–0.871, *p* = 0.011), and experiencing cardiac arrest (OR 0.542, 95% CI 0.307–0.954, *p* = 0.034) were associated with lower odds of response. Machine learning identified key predictors of response, with the Stacking Classifier achieving the highest performance (accuracy 64.5%, precision 66.6%, recall 64.5%, F1 score 61.3%).

**Conclusion:**

This study supports the potential benefits of intravenous amantadine in non-traumatic DOC-patients. Higher premorbid mRS, and pathological cerebral imaging were key predictors of non-response, offering potential avenues for patient selection and treatment customization. Findings from this study informed the design of our ongoing prospective study, which aims to further evaluate the long-term efficacy of amantadine.

## Background

Disorders of consciousness (DoC) frequently affect patients in (neurological) intensive care, significantly impairing rehabilitation and long-term outcomes (1). The pathophysiology of DoC involves diminished excitatory synaptic activity across the cerebral cortex. Recovery requires restoration of corticocortical, thalamocortical, and thalamostriatal connections to re-establish normal excitatory function (2).

Amantadine acts as an indirect dopamine agonist and N-methyl-D-aspartat (NMDA) receptor antagonist (3), modulating glutamatergic transmission, reducing excitotoxicity, and potentially enhancing neuronal recovery. Additionally, amantadine blocks nicotinic receptors, inhibits phosphodiesterase, and increases glial-cell-derived neurotrophic factor, further supporting brain function (4).

Despite its common use for DoC, the precise effects of amantadine remain not completely understood. While clinical experience with amantadine is generally positive, scientific evidence supporting its efficacy in DoC is limited, especially in non-traumatic brain injuries (4). Most studies focus on traumatic brain injury (TBI), demonstrating increased recovery rates, likely due to improved vigilance (5). A pivotal placebo-controlled trial by Giacino et al. showed amantadine’s ability to accelerate functional recovery in severe TBI during treatment. However, this benefit diminished following treatment discontinuation, leading to outcomes comparable to the control group. This regression has often been interpreted as a failure to sustain amantadine’s clinical benefits. Alternatively, it could reflect the effects of prematurely discontinuing treatment, raising the possibility that prolonged amantadine administration may be necessary to maintain improvements in functional recovery (6).

Evidence for amantadine in non-traumatic brain injuries is less robust: A retrospective study reported improved wakefulness and discharge outcomes in 42 out of 73 stroke patients (including acute ischemic stroke (AIS), intracranial cerebral hemorrhage (ICH), and subarachnoid hemorrhage (SAH)) treated with amantadine (7). Additionally, pooled data from five observational studies by Ruhl, Kuramatsu (8) suggested improved consciousness in AIS, ICH, and SAH patients treated with amantadine. However, these studies are limited by small sample sizes and retrospective designs (5, 7, 8). Furthermore, the impact of amantadine on the content of thought, which is required for full consciousness, remains uncertain. While amantadine could help to facilitate arousal and wakefulness, it may not sufficiently restore higher-order cognitive processes necessary for meaningful interactions or command-following. This distinction is crucial in DoC, as recovery of wakefulness alone may not equate to improved functional outcomes (9).

The primary aim of this study was to identify clinical factors associated with response to intravenous amantadine in patients with non-traumatic DoC. A secondary aim was to compare functional outcomes between responders and non-responders to assess whether amantadine treatment correlated with improved prognosis. Additionally, we investigated potential effects and risks of this treatment in a large real-world cohort. The results of this study serve as the basis for planning and sample size estimation of the prospective open-label study currently underway Amantadine for NeuroenhaNcement in acutE patients Study (ANNES), a phase IIb study in intensive and intermediate care unit patients (10).

## Methods

### Study design and setting

This retrospective study was conducted at the University Hospital Tübingen including all neurological ICU-patients treated with amantadine for DoC between January 2016 and June 2021. Data, including baseline information (age, sex, diagnoses, complications, medications, mechanical ventilation duration, vital parameters, clinical scores, and written documentation of clinical course) were extracted from electronic medical records [IntelliSpace Critical Care and Anesthesia (ICCA) Philips GmbH, Market DACH, Hamburg, Germany]. This study did not include a control group due to the challenges of retrospectively identifying comparable patients who did not receive amantadine. Treatment decisions were based on clinical judgment during routine care, and the absence of standardized protocols for non-treatment made it infeasible to reliably match untreated patients with similar characteristics.

The study was approved by the Ethics Committee of the University Hospital of Tübingen (560/2022BO2) and performed in accordance with the Declaration of Helsinki. Data were accessed for research purposes on December, 13, 2022. The authors had no access to any information that could identify any individual patient, either during or after data collection. Since this was a retrospective study, informed consent was not required per local Ethics Committee’s requirements.

### Patient selection

**Figure 1:**
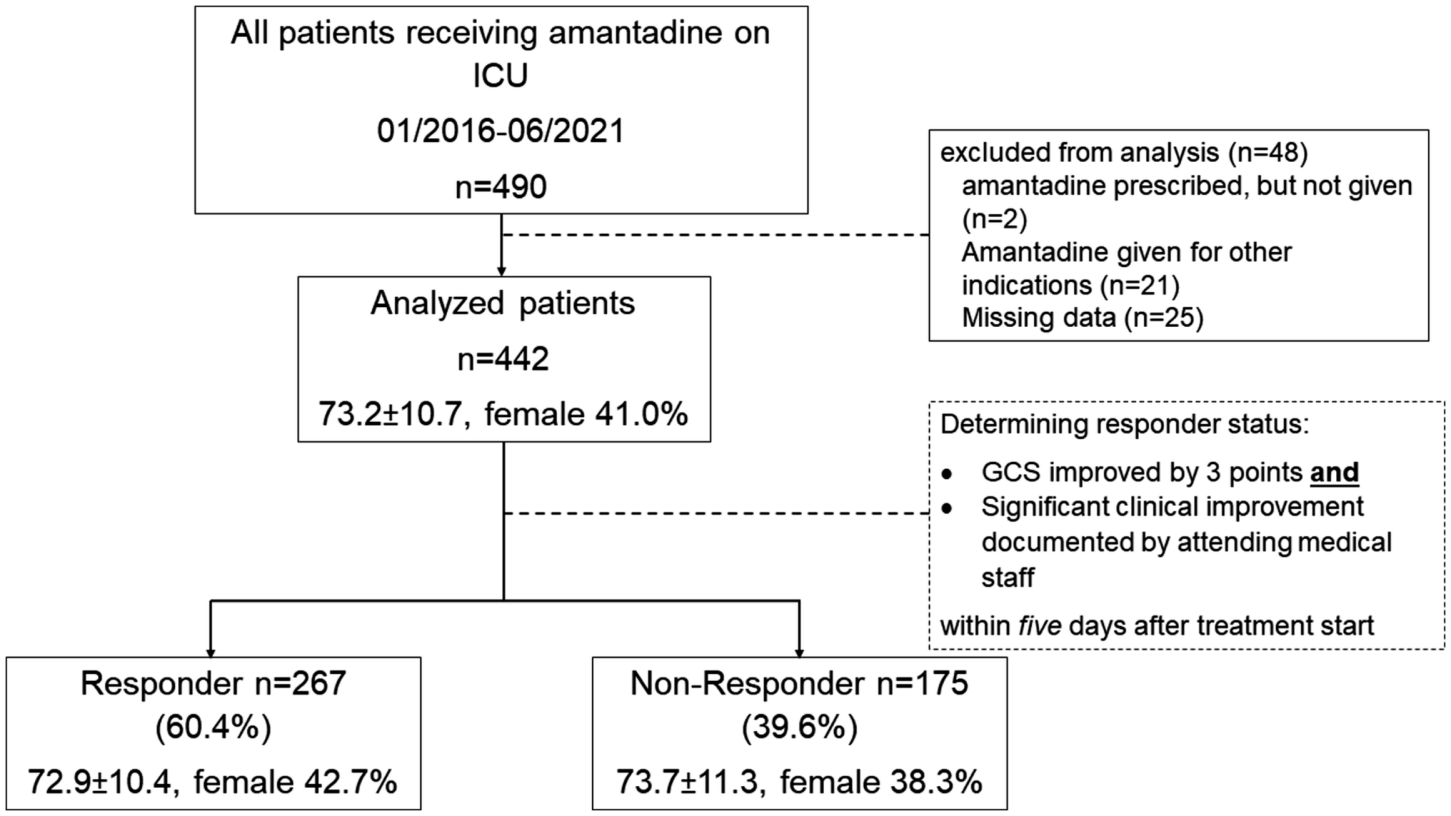
Study overview. ICU intensive care unit, GCS Glasgow Coma Scale, n number.

ICU-patients aged ≥18 years treated with amantadine for non-traumatic DoC were included (n = 490), excluding those treated for other indications (Figure 1).

### Brain disorders

Primary brain disorders included AIS, ICH, SAH, non-traumatic subdural hematoma (SDH), and meningitis/ encephalitis. Secondary brain disorders were related to systemic conditions impacting brain function, such as infection, organ insufficiencies, cardiac arrest/ resuscitation, and metabolic disorders. Combined brain disorders primary brain disorders combined with secondary causes.

### Clinical scores

The Glasgow Coma Scale (GCS) (11) was the primary measure to assess consciousness. The Richmond Agitation-Sedation Scale (RASS) (12) was used to evaluate sedation levels as part of routine ICU protocols. The Simplified Acute Physiology Score II (SAPS II) was used to evaluate the severity of ICU illness. GCS and RASS were collected during clinical routine, while SAPS-II was calculated automatically in ICCA. The National Institutes of Health Stroke Scale (NIHSS) assessed stroke severity in neurological conditions (13). Level of dependence was rated using the modified Rankin Scale (mRS). Good outcome was defined as mRS 0–2, and mortality as mRS 6. Premorbid functional status was determined using the premorbid mRS (pmRS) (14–17).

### Amantadine treatment

According to the hospital’s standard operating procedure (SOP), amantadine was administered intravenously at a daily dosage of 200–400 mg for 3–5 days (100–200 mg twice daily at 08:00 a.m. and 02:00 p.m.). Treatment was initiated only after complete weaning from sedative medications. These in-hospital guidelines were based on manufacturer guidance and prior studies, indicating a response within 3–5 days of treatment initiation (8, 18). Amantadine is approved for the treatment of vigilance reduction in post-comatose states as part of an overall therapeutic concept (18). Despite these SOP recommendations, treatment decisions, including the duration and dosage, were ultimately at the discretion of the treating physician, leading to variability in the administration of amantadine.

### Electroencephalography (EEG)

Routine EEG-recordings were performed as part of clinical work-up. EEG abnormalities were graded based on the written results (19).

### Cerebral imaging

Imaging work-up was performed as part of clinical routine, including computed tomography (CT) and/or magnetic resonance imaging (MRI) with pathological findings categorized as AIS, ICH, cerebral atrophy (diffuse, focal, global), hypoxia (hyperintensity on MRI, loss of gray-white matter differentiation on CT, or sulcal effacement on MRI/CT), microangiopathy, and old lesions (ischemic, hemorrhagic, post-traumatic, and post-surgical lesions).

### Adverse effects

Adverse effects (AE) associated with amantadine were assessed referring to the prescription form (18). Adverse events were categorized as seizures, neuropsychiatric symptoms, delirium after initiation of amantadine, and any cardiac arrhythmias during the ICU stay.

### Outcome measures

The primary outcome measure was improved consciousness, defined by an ≥3-point improvement in GCS within 5 days of amantadine treatment. Responders were defined as patients achieving this threshold, with clinical documentation reviewed to verify improvements in alertness. Secondary outcomes included RASS and SAPS II changes, GCS at discharge, good outcome (mRS 0–2) and mortality (mRS 6) at discharge as well as at follow-up. Follow-up assessments were conducted 90 days after the index event, defined as the acute illness or neurological injury through outpatient clinical visits or, in cases where direct follow-up was not feasible, via telephone interviews with patients and/or caregivers.

### Statistical analysis

Metric data were tested for normal distribution using the Kolmogorov–Smirnov test. Depending on the distribution, t-tests or Mann–Whitney *U* tests were applied for continuous variables, while nominal data were analyzed using chi-square or Fisher’s exact test. Statistical significance was defined as a *p*-value<0.05. False discovery rate (FDR) adjustments were applied to control for multiple comparisons, and Bonferroni correction was used in the multivariate logistic regression model (20–22). Pearson correlation coefficients were calculated to explore relationships between scores and responder status, with correlations interpreted as weak (0.1–0.2), moderate (0.3–0.4), or strong (≥0.5). Univariate logistic regression assessed the relationship between each independent variable and the response to amantadine treatment or other outcome parameters. Variables with statistical significance in univariate analysis (*p* < 0.05) or deemed clinically relevant were included in the multivariate logistic regression model. Linearity was assessed using the Box-Tidwell procedure (23). The absence of multicollinearity was confirmed by low correlations among predictor variables (*r* < 0.6). Outliers were evaluated using case-by-case graphs, leverage, and Cook’s distance (13–15). All statistical analyzed were performed using SPSS (version 28.0.1.1). Safety analysis included all patients, regardless of their inclusion in the primary efficacy analysis.

### Machine learning approaches to predict amantadine response

Data preprocessing included standardization, imputation of missing values using the mean for numerical columns and the most frequent value for categorical columns, and feature engineering. Key variables included age, female sex, pmRS, brain imaging and EEG pathologies, GCS, SAPS II, and etiology of DoC. Interaction terms, polynomial features, and a cumulative pathology score were used. Continuous features, such as GCS, were binned into categories (severe, moderate, mild), and categorical variables, such as sex and primary brain disorders, were one-hot encoded. Additionally, a cumulative pathology score combining imaging and EEG findings was developed to capture the overall pathology burden.

The dataset was imbalanced with respect to the target variable (responder to amantadine treatment). A RandomForestClassifier was chosen for its robustness and ability to handle both numerical and categorical data. The dataset was split (80% training, 20% testing). Numerical features were standardized using StandardScaler to ensure that they were on a comparable scale. Three base models were trained on the engineered features: Gradient Boosting, SVM, and Logistic Regression. These models were evaluated using performance metrics including accuracy, precision, recall, F1-score, confusion matrix, and classification report. To enhance model performance, two ensemble methods were employed: a Voting Classifier and a Stacking Classifier. The Voting Classifier combined the predictions of Gradient Boosting, SVM, and Logistic Regression using soft voting. The Stacking Classifier combined the predictions of the same base models using a Logistic Regression meta-classifier. Machine learning models were developed and analyzed using R (version 4.2.0).

## Results

### Demographics and clinical characteristics

The cohort included 442 patients (mean age 73.2 ± 10.7 years, 41.0% female). Amantadine treatment began on average 5.4 ± 7.2 days after beginning of weaning from mechanical ventilation, with a mean duration of 2.2 ± 2.1 days and a cumulative dosage of 900 mg (IQR 600-1200 mg).

Of the total cohort, 267 patients (60.4%) were classified as responders, and 175 (39.6%) as non-responders. Baseline characteristics, including age, sex, risk factors, brain disorders, NIHSS, GCS on admission to hospital, SAPS II on ICU-admission, were not significantly different between groups. Responders had a significantly lower median pmRS (2 [IQR 1, 2] vs. 2 [1, 3], *p* < 0.001, Cohen’s *d* = −0.37). Before treatment, responders had a lower median GCS compared to non-responders (7 [IQR 5–9] vs. 8 [IQR 5–10], *p* = 0.030, Cohen’s *d* = −0.18).

Of importance, there were no significant differences in underlying brain disorders comparing responders to non-responders, but a non-significant trend of lower incidence of cardiac arrest/ resuscitation as secondary cause for DoC in responders vs. non-responders (6.7 vs. 12%, OR 0.53, 95% CI: 0.27–1.02, *p* = 0.057).

Responders exhibited fewer pathological brain imaging findings (45.7 vs. 61.1%, OR 0.56, 95% CI:0.36–0.86, *p* = 0.008), including significantly fewer hypoxia-related changes (0.4 vs. 5.7%, *p* < 0.001), and fewer EEG abnormalities (23.6 vs. 40.0%, OR 0.47, 95% CI: 0.28–0.78, *p* = 0.003) (Table 1).

**Table 1:** Demographics and clinical characteristics of DoC cohort in ICU treated with Amantadine.

	All patients (*n* = 442)	Responder (*n* = 267)	Non-responder (*n* = 175)	*p*-value	OR	95% CI
Age (mean ± SD)	73.2 ± 10.7	72.9 ± 10.4	73.7 ± 11.3	0.443	Cohen’s *d* − 0.07	−2.90 – 1.29
Female sex, *n* (%)	181 (41.0)	114 (42.7)	67 (38.3)	0.358	1.20	0.81–1.77
pmRS (median, IQR)	2 (1, 3)	2 (1, 2)	2 (1, 3)	<0.001*	Cohen’s *d* − 0.37	
NIHSS at admission, median (IQR)	15 (6, 25)	14 (6, 25)	16 (6, 21)	0.291	Cohen’s *d* 0.14	
GCS at admission, median (IQR)	3 (3, 14)	3 (3, 14)	3 (3, 12)	0.214	Cohen’s *d* 0.08	
RASS at admission, median (IQR)	−4 (−5, −1)	−4 (−5, −1)	−4 (−5, −1)	0.454	Cohen’s *d* − 0.01	
Pre-existing dementia, *n* (%)	32 (7.2)	16 (6.0)	16 (9.1)	0.212	0.63	0.31–1.30
Pre-existing malignancy, *n* (%)	127 (28.7)	75 (28.1)	52 (29.7)	0.713	0.92	0.61–1.41
Pre-existing psychiatric comorbidity, *n* (%)	74 (16.7)	39 (14.6)	35 (20.0)	0.138	0.68	0.41–1.13
SAPS II at ICU admission (mean ± SD)	38.4 ± 14.5	38.0 ± 14.9	38.9 ± 14.0	0.269	Cohen’s d − 0.06	
Cerebral imaging pathologic, *n* (%)	229 (51.8)	122 (45.7)	107 (61.1)	0.008	0.56	0.36–0.86
AIS*	76 (17.2)	39 (14.6)	37 (21.1)	0.145	0.69	0.41–1.14
ICH*	86 (19.5)	41 (17.6)	39 (22.3)	0.389	0.80	0.50–1.31
Cerebral atrophy* (diffuse/focal/global)	16 (3.6)	7 (2.6)	9 (5.1)	0.216	0.53	0.19–1.46
Microangiopathy*	43 (9.7)	26 (9.7)	17 (9.7)	0.799	1.09	0.57–2.08
Lesions* (chronic ischemic/hemorrhagic changes/post-traumatic lesions, post-surgical reasons)	36 (8.1)	26 (9.7)	10 (5.7)	0.082	1.94	0.91–4.16
EEG pathologic, *n* (%)	133 (30.1)	63 (23.6)	70 (40.0)	0.003*	1.4	0.57–3.46
Mechanical ventilation, *n* (%)	428 (96.8)	264 (98.9)	164 (93.7)	0.002*	5.90	1.62–21.47
Mechanical ventilation duration [days] (mean ± SD, 95% CI)	16.9 ± 16.2	16.3 ± 16.3 14.36–18.28	17.8 ± 16.0 15.46–20.22	0.336	Cohen’s d − 0.09	
EVD, *n* (%)	50 (11.3)	30 (11.2)	20 (11.4)	0.950	1.06	0.57–1.98
ICP, *n* (%)	51 (11.5)	30 (11.2)	21 (12.0)	0.806	0.92	0.51–1.68
Primary brain disorders, *n* (%)	237 (53.6)	139 (52.1)	98 (56.0)	0.418	0.85	0.58–1.25
AIS*, *n* (%)	148 (33.5)	86 (32.2)	62 (35.4)	0.484	0.87	0.58–1.29
ICH*, *n* (%)	60 (13.6)	32 (12.0)	28 (16.0)	0.229	0.71	0.41–1.24
SAH*, *n* (%)	55 (12.4)	32 (12.0)	23 (13.1)	0.719	0.90	0.51–1.60
Non-traumatic SDH*, *n* (%)	28 (6.3)	13 (4.9)	15 (8.6)	0.119	0.55	0.25–1.18
Meningitis/Encephalitis*, *n* (%)	6 (1.4)	3 (1.1)	3 (1.7)	0.601	0.65	0.13–3.27
Primary brain disorders combined with secondary causes for DoC, *n* (%)	170 (38.5)	101 (37.8)	69 (39.4)	0.736	0.93	0.63–1.38
Secondary causes for DoC, *n* (%)	205 (46.4)	128 (47.9)	77 (44.0)	0.463	1.17	0.80–1.72
Sepsis*, *n* (%)	98 (22.2)	57 (21.3)	41 (23.4)	0.608	0.89	0.56–1.40
Cardiac arrest*, *n* (%)	39 (8.8)	18 (6.7)	21 (12.0)	0.057	0.53	0.27–1.02
ARDS*, *n* (%)	137 (31.0)	86 (32.2)	51 (29.1)	0.496	1.16	0.76–1.75
Other diseases*, *n* (%)	87 (19.7)	57 (21.3)	30 (17.1)	0.321	1.28	0.78–2.10

AIS, acute ischemic stroke; ICH, intracerebral hemorrhage; SAH, subarachnoidal hemorrhage; SDH, subdural hematoma; DoC, disorders of consciousness; ARDS, acute respiratory distress syndrome; EVD, extraventricular drainage; ICP, intracranial pressure; GCS, Glasgow Coma Scale; RASS, Richmond Agitation and Sedation Scale; SAPS II, Simplified Acute Physiology Score II; IQR, Inter Quartile Range; mRS, modified rankin scale; NIHSS, National Institute of Health Stroke Scale; OR, odds ratio. *Multiple diagnoses possible.

### Treatment response

After treatment initiation, responders demonstrated significantly higher median GCS on day 1 (10 [IQR 7–11] vs. 9 [IQR 5–11], *p* < 0.001, Cohen’s *d* = 0.37), higher median RASS (−2 [IQR −3, −1] vs. -3 [IQR –4, −1], *p* = 0.002, Cohen’s *d* = 0.21), and lower SAPS II (31.0 ± 11.4 vs. 35.3 ± 12.0, *p* < 0.001, Cohen’s *d* = −0.36).

**Figure 2:**
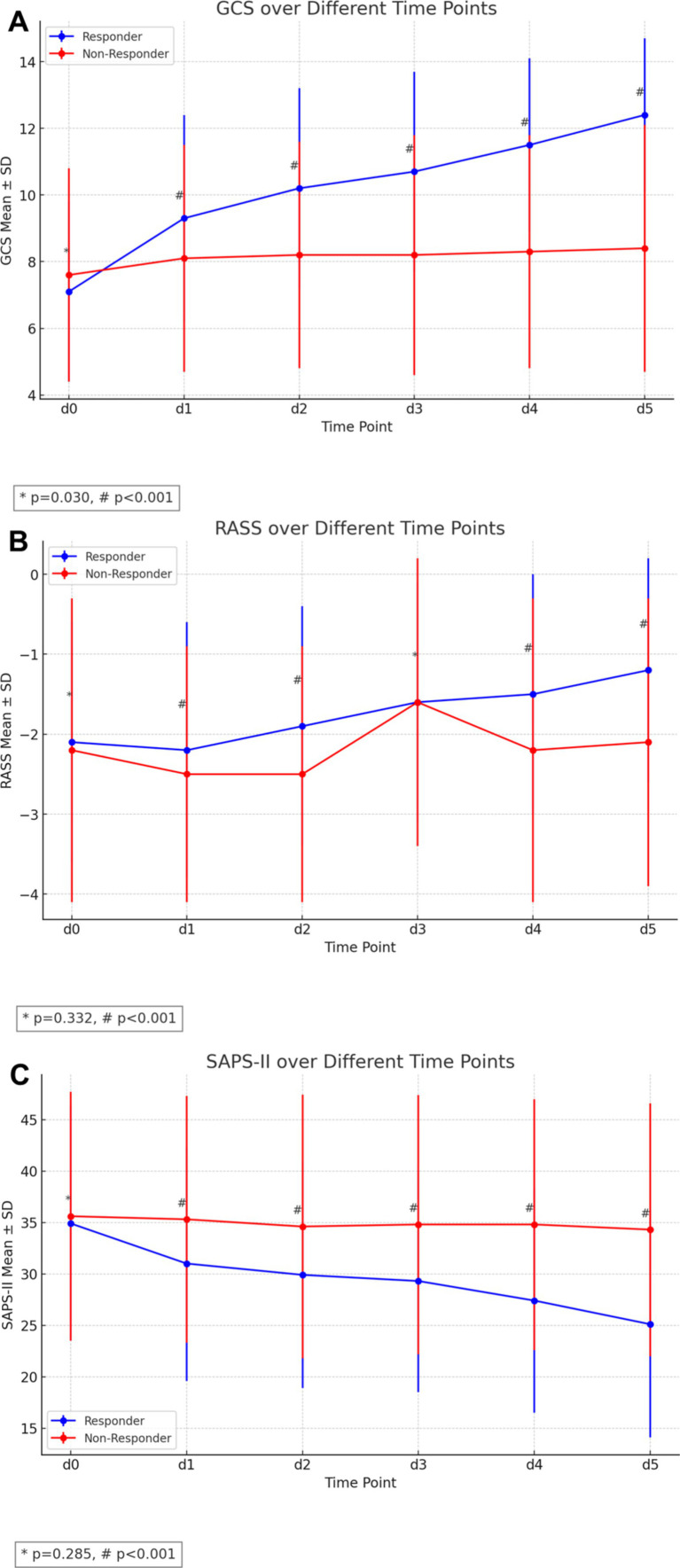
Clinical outcomes of amantadine treatment in ICU-patients with non-traumatic disorders of consciousness. **(A)** Median GCS scores over the first 5 days of treatment, showing a significant improvement in responders compared to non-responders. **(B)** Displays the mRS at different time points. **(C)** Depicts SAPS-II.

These trends persisted on subsequent days, with responders consistently showing better scores after beginning of amantadine treatment (Figures 2A–C and Table 2). Weaning from mechanical ventilation occurred earlier in responders (4.7 ± 6.6 vs. 6.5 ± 7.9 days, *p* = 0.014, Cohen’s *d* = −0.25).

**Table 2:** Treatment course of DoC cohort treated with Amantadine: GCS, RASS and SAPS II.

	All patients (*n* = 442)	Responder (*n* = 267)	Non-Responder (*n* = 175)	*p*-value	Cohen’s *d*
GCS on d0 (before amantadine initiation), median (IQR)	8 (5, 9)	7 (5, 9)	8 (5, 10)	0.030*	−0.18
RASS on d0 (before amantadine initiation), median (IQR), d0	−2 (−3, −1)	−2 (−3, −1)	−2 (−4, −1)	0.332	−0.19
SAPS II on d0 (before amantadine initiation), (mean ± SD)	35.2 ± 11.7	34.9 ± 11.4	35.6 ± 12.1	0.285	−0.06
GCS on d1, median (IQR)	9 (6, 11)	10 (7, 11)	9 (5, 11)	<0.001*	0.37
RASS on d1, median (IQR)	−2 (−4, −1)	−2 (−3, −1)	−3 (−4, −1)	0.002*	0.21
SAPS II on d1 (mean ± SD)	32.7 ± 11.8	31.0 ± 11.4	35.3 ± 12.0	<0.001*	−0.36
GCS on d2, median (IQR)	10 (7, 12)	11 (9, 13)	9 (5, 11)	<0.001*	0.61
RASS on d2, median (IQR)	−2 (−4, −1)	−2 (−3, −1)	−3 (−4, −1)	<0.001*	0.36
SAPS II on d2 (mean ± SD)	31.7 ± 11.9	29.9 ± 11.0	34.6 ± 12.8	<0.001*	−0.40
GCS on d3, median (IQR)	10 (8, 13)	11 (9, 13)	9 (5, 11)	<0.001*	0.78
RASS on d3, median (IQR)	−2 (−3, −1)	−1 (−3, −1)	−2 (−4, −1)	0.497	−0.00
SAPS II on d3 (mean ± SD)	30.9 ± 12.0	29.3 ± 10.8	34.8 ± 12.6	<0.001*	−0.56
GCS on d4, median (IQR)	11 (9, 13)	11 (10, 14)	9 (5, 11)	<0.001*	1.08
RASS on d4, median (IQR)	−1 (−3, −1)	−1 (−2, 0)	−2 (−4, −1)	<0.001*	0.47
SAPS II on d4 (mean ± SD)	30.2 ± 12.0	27.4 ± 10.9	34.8 ± 12.2	<0.001*	−0.65
GCS on d5, median (IQR)	11 (9, 14)	13 (11, 14)	9 (5, 11)	<0.001*	1.38
RASS on d5, median (IQR)	−1 (−3, 0)	−1 (−2, 0)	−2 (−4, −1)	<0.001*	0.58
SAPS II on d5 (mean ± SD)	29.3 ± 12.1	25.1 ± 11.0	34.3 ± 12.3	<0.001*	−0.71

GCS, Glascow Coma Scale; RASS, Richmond Agitation and Sedation Scale; SAPS II, Simplified Acute Physiology Score II; IQR, Inter Quartile Range.

### Primary outcomes

Responders had a significantly lower median mRS at discharge (5 [IQR 4–5] vs., 5 [IQR 5–6], *p* < 0.001, Cohen’s *d* = −0.47), with a higher proportion achieving good outcomes at follow-up (4.9 vs. 1.1%, OR 6.14, 95% CI: 1.35–28.01, *p* = 0.004). Mortality was significantly lower in responders both at discharge (13.5 vs. 27.4%, OR 0.41, 05% CI: 0.25–0.67, *p* < 0.001) and at follow-up (16.9 vs. 32.0%, OR 0.43, 95% CI: 0.24–0.77, *p* = 0.002) (Table 3).

**Table 3:** Outcome parameters of DoC cohort treated with Amantadine.

	All patients (*n* = 442)	Responder (*n* = 267)	Non-responder (*n* = 175)	*p*-value	OR	95% CI
Discharge						
Hospital stay [days] (mean ± SD) Missing value = 0	26.5 ± 21.2	25.7 ± 20.6	27.7 ± 22.0	0.330	Cohen’s d − 0.09	
mRS, median (IQR) Missing value = 4	5 (5, 5)	5 (4, 5)	5 (5, 6)	<0.001*	Cohen’s d − 0.47	
Mortality, *n* (%) Missing values = 0	84 (19.0)	36 (13.5)	48 (27.4)	<0.001*	0.41	0.25–0.67
GCS, median (IQR) Missing values = 108	14.0 (11, 15)	14.0 (13, 15)	12.0 (9, 14)	<0.001*	Cohen’s d 0.88	
Follow-up 90 days						
mRS, median (IQR) Missing values = 225	6 (4, 6)	5 (3, 6)	6 (5, 6)	<0.001*	Cohen’s d − 0.57	
Good outcome, *n* (%) Missing values = 225	15 (3.4)	13 (4.9)	2 (1.1)	0.004*	6.14	1.35–28.01
Mortality, *n* (%) Missing values = 225	101 (22.9)	45 (16.9)	56 (32.0)	0.002*	0.43	0.24–0.77

GCS, Glasgow Coma Scale; mRS, modified rankin scale; NIHSS, National Institute of Health Stroke Scale.

### Complications and secondary outcomes

No significant differences were observed in the overall complication rate between responders and non-responders (27.7 vs. 31.4%, RR 0.89, 95% CI: 0.66–1.18, *p* = 0.402). Seizures, categorized as occurring after amantadine initiation, were more frequent in non-responders (10.1 vs. 16.6%, OR 0.48, 95% CI: 0.29–0.81, *p* = 0.046). Responders experienced a higher incidence of delirium (33.0 vs. 22.3%, OR 1.47, 95% CI: 1.06–2.03, *p* = 0.015). Any cardiac arrythmias during ICU-stay occurred in 37.8 vs. 33.7% (*p* = 0.38) (Table 4).

**Table 4:** Clinical treatment course of the DoC cohort: details of Amantadine treatment and clinical complications during ICU stay.

	All patients (*n* = 442)	Responder (*n* = 267)	Non-responder (*n* = 175)	*p*-value	RR	95% CI
Amantadine treatment: duration [days] (mean ± SD)	2.2 ± 2.1	2.3 ± 2.1	2.1 ± 2.0	0.512	Cohen’s *d* 0.06	
Amantadine: cumulative dosage [mg] (mean ± SD)	907.2 ± 819.2	927.0 ± 762.7	877.1 ± 886.0	0.529	Cohen’s *d* 0.06	
Begin of amantadine treatment regarding weaning off from mechanical ventilation (mean ± SD)	5.4 ± 7.2	4.7 ± 6.6	6.5 ± 7.9	0.014*	Cohen’s *d* − 0.25	
Possible complications due to amantadine treatment, *n* (%)	129 (29.2)	74 (27.7)	55 (31.4)	0.402	0.89	0.66–1.18
Epileptic seizure during ICU stay, *n* (%)	56 (12.7)	27 (10.1)	29 (16.6)	0.046*	0.48	0.29–0.81
Cardiac arrythmia during ICU stay, *n* (%)	160 (36.2)	101 (37.8)	59 (33.7)	0.380	1.12	0.87–1.45
Myocardial infarction, n (%)	124 (28.1)	75 (28.1)	49 (28.0)	0.984	1.00	0.74–1.36
Infection, *n* (%)	362 (81.9)	211 (79.0)	151 (86.3)	0.053	0.92	0.84–1.00
Acute/chronic renal insufficiency, *n* (%)	230 (52.0)	143 (53.6)	87 (49.7)	0.430	1.08	0.89–1.30
Dialysis, *n* (%)	4 (0.9%)	2 (0.7)	2 (1.1)	0.670	0.66	0.11–3.75
Endocrinological disorder, *n* (%)	45 (10.2)	28 (10.5)	17 (9.7)	0.793	1.07	0.61–1.88
Electrolyte imbalances, *n* (%)	92 (21.5)	56 (21.0)	39 (22.3)	0.743	0.94	0.66–1.35
Elevated liver enzymes, *n* (%)	116 (26.2)	75 (28.1)	41 (23.4)	0.277	1.19	0.86–1.66
Gastrointestinal complications, *n* (%)	76 (17.2)	46 (17.2)	30 (17.1)	0.981	1.00	0.66–1.52
ARDS, *n* (%)	237 (53.6)	147 (55.1)	90 (51.4)	0.456	1.07	0.89–1.28
Delirium during ICU stay, *n* (%)	127 (28.7)	88 (33.0)	39 (22.3)	0.015*	1.47	1.06–2.03

ARDS, acute respiratory distress syndrome; ICU intensive care unit.

### Correlation analysis

Significant correlations were observed between GCS and SAPS II scores, with higher GCS scores associated with lower SAPS II scores (*r* = −0.643, *p* < 0.001). Moderate correlations were found between RASS and both GCS and SAPS II. Strong negative correlations were observed between GCS scores and mRS at discharge, indicating that better neurological function was associated with less disability. Similar negative correlations between RASS and mRS at discharge suggested that less sedation or agitation was linked to better functional outcomes. Positive correlations between SAPS II and mRS indicated that greater illness severity predicted worse functional outcomes.

Additionally, GCS scores were strongly negatively correlated with in-hospital mortality, suggesting that higher GCS scores were associated with a lower likelihood of death. Negative correlations between RASS scores and in-hospital mortality indicated that better RASS scores were linked to reduced mortality risk, while positive correlations between SAPS II and mortality showed that greater illness severity was associated with higher mortality rates.

### Multivariate logistic regression for outcomes at discharge and follow-up

**Figure 3:**
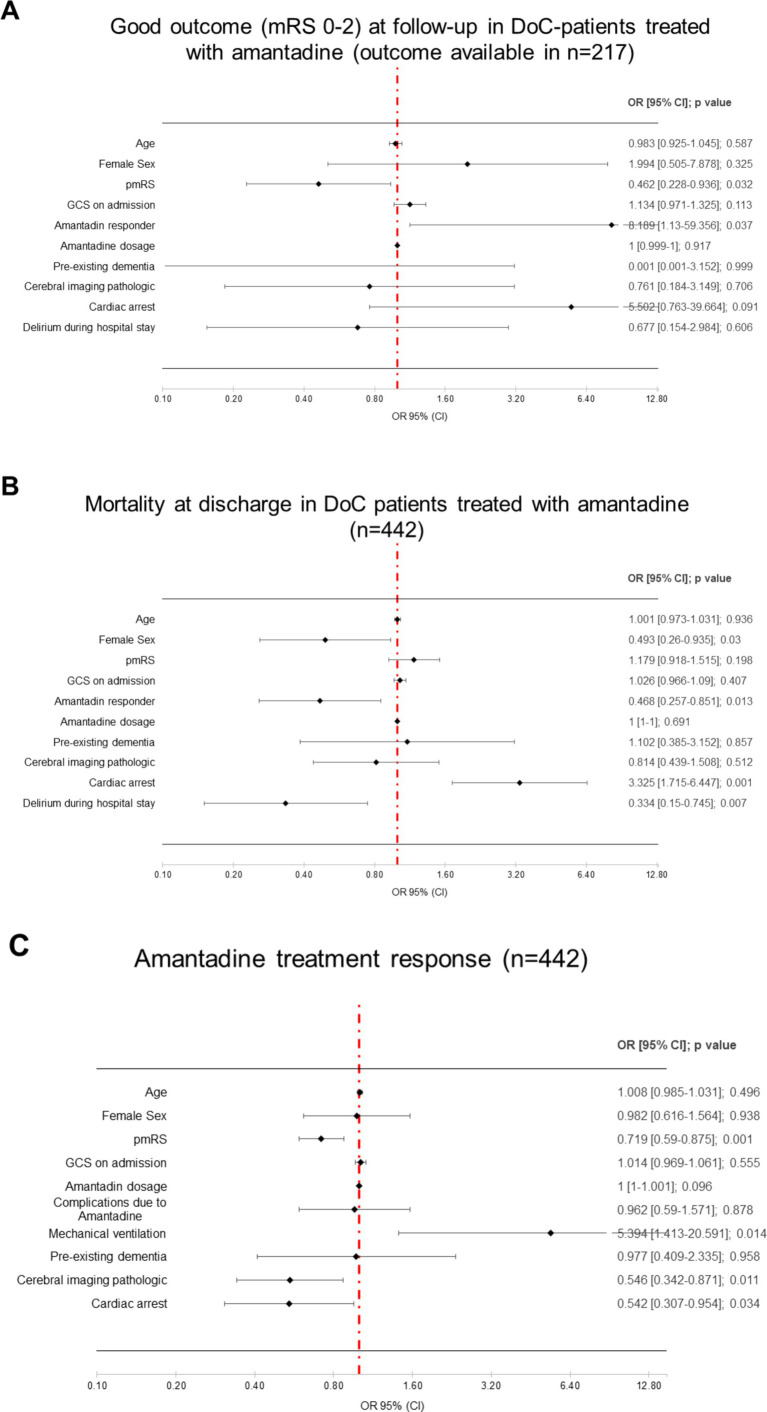
Multivariate logistic regression for mortality at discharge **(A)**, good outcome at follow-up **(B)**, and amantadine treatment response **(C)**. GCS, Glascow Coma Scale; CI, confidence interval; OR, odds ratio; pmRS, premorbid modified rankin scale.

Multivariate logistic regression revealed that responders had significantly lower odds of in-hospital mortality (OR 0.468, 95% CI: 0.257–0.851, *p* = 0.013), while experiencing cardiac arrest increased mortality risk (OR 0.334, 95% CI: 0.150–0.745, *p* = 0.007). Females had significantly lower mortality odds compared to males (OR 0.493. 95% CI: 0.260–0.935, *p* = 0.030). Other factors, including age, pmRS, GCS at admission, total amantadine dosage, pre-existing dementia, and pathological imaging results, did not significantly affect mortality (Figure 3A).

For good outcomes at follow-up, higher pmRS was associated with lower odds of achieving a good outcome (OR 0.740, 95% CI: 0.630–0.869, *p* < 0.001), while being a responder to amantadine was significantly increased the odds of good outcome (OR 8.189, 95% CI: 1.130–59.356, *p* = 0.037) (Figure 3B).

### Univariate and multivariate logistic regression for treatment response

In univariate analysis, higher pmRS (OR 0.740, 95% CI: 0.630–0.869, *p* < 0.001), and pathological brain imaging (OR 0.558, 95% CI: 0.363–0.860, *p* = 0.008) were associated with a lower likelihood of response to amantadine (Table 5).

**Table 5:** Univariate logistic regression for amantadine treatment response.

Variable	OR	95% CI	*p*-value
Age	0.993	0.975–1.011	0.443
Female sex	1.201	0.814–1.773	0.357
pmRS	0.740	0.630–0.869	<0.001
GCS at admission	1.015	0.978–1.054	0.426
Pathological EEG	1.400	0.567–3.457	0.466
Pathological brain imaging	0.558	0.363–0.860	0.008*
EVD	0.981	0.538–1.789	0.950
Pre-existing dementia	0.633	0.308–1.303	0.214
Pre-existing psychiatric diagnoses	0.684	0.414–1.131	0.139
Primary disorders of brain	0.841	0.573–1.233	0.374
Cardiac arrest/resuscitation	0.633	0.389–1.030	0.066
AIS	0.866	0.579–1.295	0.483
ICH	0.715	0.414–1.236	0.230
SAH	0.868	0.488–1.545	0.631
non-traumatic SDH	0.546	0.253–1.177	0.123
Brain swelling	1.402	0.702–2.798	0.338
Secondary causes of DoC	1.011	0.686–1.490	0.958
Mechanical ventilation	5.902	1.623–21.472	0.007*
Cumulative amantadine dosage [mg]	1.000	1.000–1.000	0.530

pmRS: premorbid modified Rankin Scale, EEG: electroencephalography, EVD: extraventricular drainage, AIS: acute ischemic stroke, ICH: intracerebral hemorrhage, SAH: subarachnoid hemorrhage, DoC: disorders of consciousness, *p significant.

In multivariate analysis, higher pmRS remained a significant predictor of lower response odds (OR 0.719, 95% CI 0.590–0.875, *p* < 0.001), while pathological brain imaging (OR 0.546, 95% CI 0.342–0.871, *p* = 0.011), and cardiac arrest (OR 0.542, 95% CI 0.307–0.954, *p* = 0.034) also reduced the likelihood of response (Figure 3C).

### Machine learning models for prediction of treatment response

The Gradient Boosting Classifier achieved an accuracy of 58.1%, a precision of 55.2%, a recall of 58.1%, and an F1 score of 53.7%. The SVM Classifier achieved an accuracy of 55.9%, a precision of 54.6%, a recall of 55.9%, and an F1 score of 53.9%. The Logistic Regression model showed slightly better performance with an accuracy of 59.1%, a precision of 56.3%, a recall of 59.1%, and an F1 score of 53.3%.

**Figure 4:**
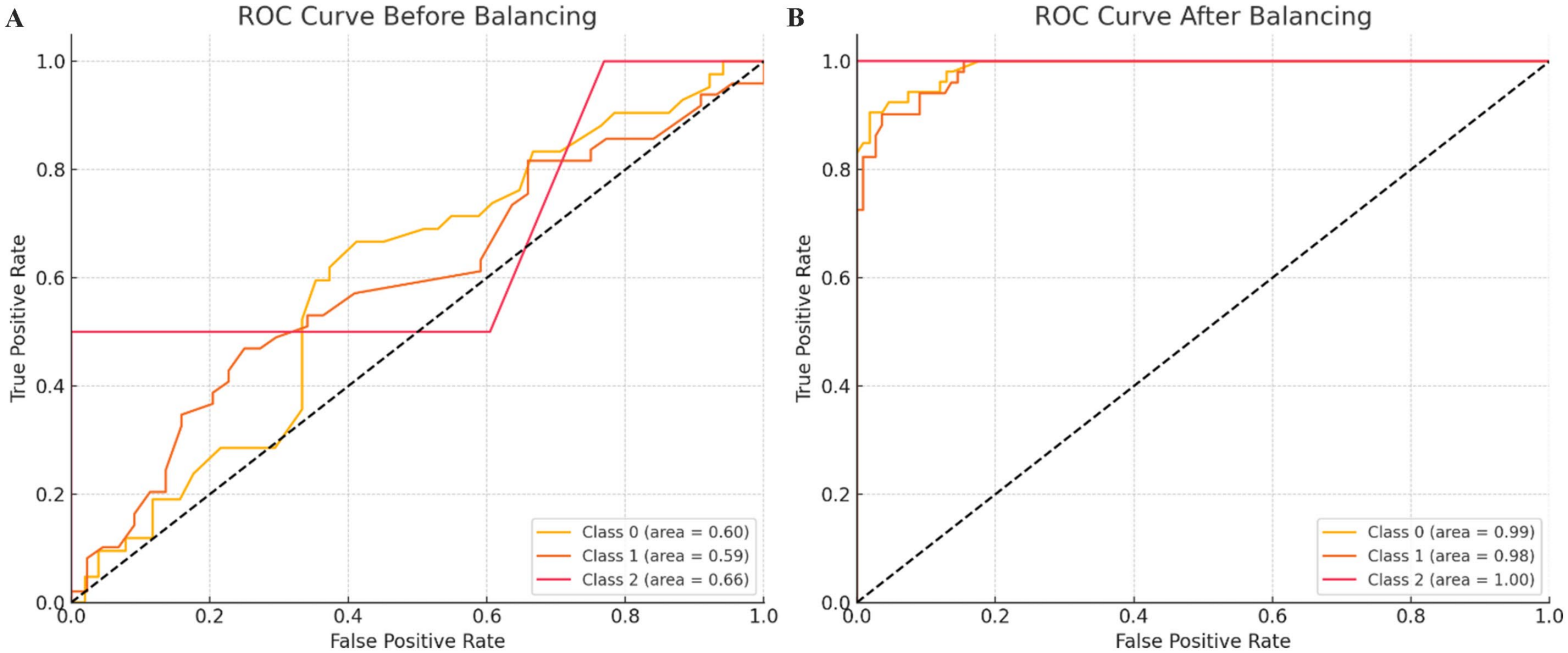
ROC curves for the model. The ROC curves before and after balancing the dataset provide a visual representation of the model’s performance in distinguishing between classes. The ROC curve before balancing **(A)** the dataset shows the true positive rate (sensitivity) against the false positive rate (1–specificity) for each class: Class 0: Area Under the Curve (AUC) = 0.60, Class 1: AUC = 0.79, and Class 2: AUC = 1.00. The ROC curve after balancing **(B)** the dataset shows the improved true positive rate against the false positive rate for each class: Class 0: AUC = 0.97, Class 1: AUC = 0.94, and Class 2: AUC = 1.00.

Ensemble models improved overall performance. The Voting Classifier achieved an accuracy of 58.1%, a precision of 57.1%, a recall of 58.1%, and an F1 score of 55.76%. The confusion matrix for the Voting Classifier was [[16, 26, 0], [11, 38, 0], [0, 2, 0]]. The Stacking Classifier demonstrated the best overall performance, with an accuracy of 64.5%, a precision of 66.6%, a recall of 64.5%, and an F1 score of 61.3%. The confusion matrix for the Stacking Classifier was [[16, 26, 0], [5, 44, 0], [0, 2, 0]]. In summary, the Stacking Classifier outperformed the other models, showing the highest accuracy and F1 score. Ensemble methods, particularly the Stacking Classifier, improved performance over individual models, especially in handling majority classes. However, all models struggled with predicting the minority class (Class 2), indicating the need for further balancing or additional feature engineering. Balancing the dataset significantly improved the model’s accuracy, precision, recall, and F1-score. This was also shown by the higher AUC values. The confusion matrices and classification reports illustrated that the balanced model performed well across all classes, including the previously underrepresented class 2 (Figure 4).

## Discussion

We conducted a retrospective analysis of 442 ICU-patients receiving intravenous amantadine for non-traumatic DoC from January 2016 to June 2021 to evaluate treatment effects, safety, outcomes, and potential predictors of response. Our main findings are as follows:

Firstly, 60.4% of patients responded to amantadine, with responders consistently having better GCS and RASS, despite starting with lower GCS before treatment initiation. SAPS II scores were lower in responders throughout the treatment period, despite comparable baseline severity.

Secondly, responder status was associated with better outcomes. Responders had significantly lower in-hospital mortality, and the survival benefit persisted at follow-up. At follow-up, a higher proportion of responders achieved functional independence, indicating meaningful recovery in some patients. However, hospital stay was not significantly shortened. The lack of significant differences in baseline characteristics, such as age, sex, and comorbidities, strengthens our findings and could suggest that differences in outcomes could be attributable to amantadine treatment response.

Thirdly, multivariate analysis identified higher pmRS, pathological imaging, and cardiac arrest as predictors of non-response. Age, pmRS, GCS at admission, total amantadine dosage, and pre-existing dementia did not significantly predict outcomes. Female sex and delirium were associated with lower mortality, while cardiac arrest predicted higher mortality.

Several studies have investigated amantadine’s effect in both traumatic and non-traumatic DoC, with mixed findings. Giacino and Whyte demonstrated faster improvement in patients with severe TBI receiving amantadine, though effects diminished after discontinuation (6). Similarly, Ghalaenovi, Fattahi (24) reported initial GCS improvement within 7 days in TBI patients, but no long-term differences in GCS, DRS, Karnofsky Performance Index and Mini-Mental State Examination at 6 months. Nekrasova, Kanarskii (25) found better survival in younger patients with less impaired consciousness (measured by the CRS-R), while hypoxic brain injury predicted poorer prognosis.

In non-traumatic conditions, amantadine showed a positive response in approximately 70% of stroke cases (26), with effects sustained up to 2 months (5). Leclerc and Riker reported a 53% response rate, but no reduction in hospital stay or improved survival (7). Ruhl, Kuramatsu (8) found improved consciousness within 5 days of starting amantadine in a pooled analysis of five single-center studies involving patients with various non-traumatic conditions such as AIS, ICH, SAH, meningitis, and status epilepticus, but survival rates at 3-month follow-up were unchanged. Additionally, Zorowitz, Smout (27) found no significant impact of amantadine on motor recovery or discharge outcomes in stroke rehabilitation patients. These studies suggest amantadine may provide short-term improvements in consciousness, but its long-term effects on survival, functional and cognitive recovery, and rehabilitation remain uncertain.

Fourthly, complications occurred in 30% of both responders and non-responders, exceeding rates reported in the prescription form (18). Non-responders experienced more seizures after amantadine initiation, while responders had higher rates of delirium and any cardiac arrhythmias during ICU stay. Previous studies, such as Giacino, Whyte (6) reported lower rates of seizures (4.5%) and cardiac issues (14%), with agitation and insomnia in 14%. This discrepancy may stem from the retrospective nature of our analysis and the challenges in distinguishing between amantadine-related side effects and complications related to the ICU-setting. Our high rate of cardiac arrhythmia might be a result of including all types of cardiac arrhythmias (mostly atrial fibrillation but also ventricular arrhythmias) coded in the medical record during the ICU-stay, and not only after amantadine initiation. Cardiac arrhythmias may relate to amantadine’s autonomic effects (28), underscoring the need for close cardiovascular monitoring in future studies (e.g., our ongoing trial NCT05479032(10)). Delirium, more common in responders, could result from amantadine’s dopaminergic and NMDA receptor antagonistic effects during reafferentation (2, 3, 29, 30). Maldonado et al. highlighted dopamine and glutamate imbalances as drivers of delirium, with excess release of these neurotransmitters implicated in cognitive and behavioral changes (31, 32). Additionally, hyperactive delirium could result in higher GCS, leading to an overrepresentation of delirium among responders.

Lastly, our machine learning models identified key predictors of response, such as lower pmRS, imaging, and EEG findings. The Stacking Classifier achieved an accuracy of 64.5%, suggesting that combining clinical, imaging, and EEG data may guide personalized amantadine treatment. However, these models require validation in prospective studies including standardized interpretation of imaging and EEG findings. Our study is the largest retrospective analysis of real-world data on amantadine for non-traumatic DoC and the first to investigate predictors of response, providing real-world insights across divers ICU settings. Additionally, our machine learning models present a novel approach to identifying predictors of treatment response, laying potential groundwork for future personalized treatment strategies. However, inherent limitations of the retrospective design must be acknowledged. These include selection bias, variability in record-keeping quality, the absence of a control group, and the reliance on routine clinical documentation for outcomes. Variability in amantadine administration, despite in-hospital recommendations, and interpretation of EEG and imaging findings reflects the challenges of real-world ICU practice, as clinical findings that might influence prognosis could have been under- or over-reported. For responder classification, we relied on GCS and clinical documentation, as other outcome measures [e.g., Disability Rating Scale (DRS), Full Outline of UnResponsiveness Score (FOUR), Coma Recovery Scale-Revised (CRS-R) were not routinely used in clinical practice]. Furthermore, this design precludes the collection of detailed information on all potential AE, particularly in an ICU setting. Furthermore, missing long-term follow-up data on survival and cognitive outcomes may have introduced bias, as loss to follow-up could be influenced by the patients’ clinical condition. These limitations underscore the importance of prospective studies, as our findings in the retrospective study provided valuable insights for the design of the ongoing prospective open-label study with amantadine (ANNES), which includes a robust framework for determining sample size, intervention protocols, and responder definitions (10).

## Conclusion

Overall, our study highlights the potential benefits of amantadine for patients with non-traumatic DoC while emphasizing the need for prospective validation. Future studies should refine treatment protocols, integrate predictive algorithms, and explore biomarkers to improve outcomes and optimize patient selection. Our ongoing prospective open-label study [ANNES (10)], informed by the results of this study, will play a crucial role in addressing knowledge gaps.

## Data Availability

The raw data supporting the conclusions of this article will be made available by the authors, without undue reservation.
